# Development and external validation of a machine learning model for the prediction of persistent acute kidney injury stage 3 in multi-centric, multi-national intensive care cohorts

**DOI:** 10.1186/s13054-024-04954-8

**Published:** 2024-06-04

**Authors:** Simone Zappalà, Francesca Alfieri, Andrea Ancona, Fabio Silvio Taccone, Riccardo Maviglia, Valentina Cauda, Stefano Finazzi, Antonio Maria Dell’Anna

**Affiliations:** 1 U-Care Medical srl, Corso Castelfidardo 30A, 10129 Turin, Italy; 2 Department of Intensive Care, Hôpital Universitaire de Bruxelles (HUB), Université Libre de Bruxelles (ULB), Route de Lennik 808, 1070 Brussels, Belgium; 3 Department of Anesthesia, Intensive Care and Emergency Medicine, Fondazione Policlinico Universitario Agostino Gemelli IRCCS, 00168 Rome, Italy; 4 Department of Applied Science and Technology, Politecnico di Torino, C.so Duca degli Abruzzi 24, 10129 Turin, Italy; 5 Clinical Data Science Laboratory, Istituto di Ricerche Farmacologiche Mario Negri IRCCS, Via Stezzano 87, 24126 Bergamo, BG Italy

**Keywords:** Artificial intelligence, Acute kidney injury, Biomarker, Intensive care unit

## Abstract

**Background:**

The aim of this retrospective cohort study was to develop and validate on multiple international datasets a real-time machine learning model able to accurately predict persistent acute kidney injury (AKI) in the intensive care unit (ICU).

**Methods:**

We selected adult patients admitted to ICU classified as AKI stage 2 or 3 as defined by the “*Kidney Disease: Improving Global Outcomes*” criteria. The primary endpoint was the ability to predict AKI stage 3 lasting for at least 72 h while in the ICU. An explainable tree regressor was trained and calibrated on two tertiary, urban, academic, single-center databases and externally validated on two multi-centers databases.

**Results:**

A total of 7759 ICU patients were enrolled for analysis. The incidence of persistent stage 3 AKI varied from 11 to 6% in the development and internal validation cohorts, respectively and 19% in external validation cohorts. The model achieved area under the receiver operating characteristic curve of 0.94 (95% CI 0.92–0.95) in the US external validation cohort and 0.85 (95% CI 0.83–0.88) in the Italian external validation cohort.

**Conclusions:**

A machine learning approach fed with the proper data pipeline can accurately predict onset of Persistent AKI Stage 3 during ICU patient stay in retrospective, multi-centric and international datasets. This model has the potential to improve management of AKI episodes in ICU if implemented in clinical practice.

**Supplementary Information:**

The online version contains supplementary material available at 10.1186/s13054-024-04954-8.

## Background

Acute kidney injury (AKI) is a common complication during acute critical illness, which is estimated to affect one in two patients admitted to the intensive care unit (ICU) [1] exhibiting an increasing incidence over the past decade [2]. Li et al. [3] argued that AKI is often underdiagnosed and undertreated, despite its common occurrence, resulting in a potential increase in the risk of in-hospital mortality.

In the last years, researchers have focused on AKI episodes that do not resolve within 48 h. The Acute Disease Quality Initiative (ADQI) 16 Workgroup defined this condition as persistent AKI, in opposition to complete renal recovery within a brief time, referred as transient AKI. This distinction is clinically relevant since AKI episodes present different outcomes according to their duration. Indeed, patients without renal recovery within 3–7 days showed reduced survival over the following year (40% vs 90%) and increased risk of developing chronic renal disease than those who recovered renal function [4]. Early identification of patients at risk of persistent AKI would allow a more accurate risk stratification and individualized management [5, 6]. Nonetheless, 48 h time-frame has been chosen by guidelines to warrant prompt clinical intervention, but stage 3 AKI lasting more than 72 h (p-AKI3) may be a better definition of non-resolving AKI, eventually requiring renal replacement therapy.

Due to the various aetiologies of AKI and the complex mechanisms behind renal dysfunction, traditional biological indicators or renal-specific biomarkers proved poor performance to predict pAKI 3 [7, 8]. Experimental studies suggested that, because of different existing injury pathways, multiple biomarkers may be necessary in this context [9]. As such, a variety of measurement dedicated to the early prediction of pAKI 3 have been proposed, including urinary parameters, renal ultrasonography, estimates of glomerular filtration rate and biomarkers of kidney damage. Although predicting short-term recovery might help in optimizing patient management and anticipate outcome, available imaging tests, biomarkers, and scores have not yet been validated and, at best, were found to be poorly efficient in preliminary studies [10].

A novel approach to predict pAKI3 could involve the use of Artificial Intelligence (AI) and Machine Learning (ML), that could assess clinical data and predict the severity and duration of AKI episodes, overcoming the traditional biomarker approaches [11], more expensive and time-consuming.

ML techniques have been already developed and externally validated to predict AKI stage 2 or 3 within 24 h [12] or AKI stage 3 within 48 h [13]. To our knowledge, the only works that focused on the prediction of pAKI3 focused only on septic patients [14] or post postoperative patients [15]; these models are still not externally validated. Our approach focused on a novel set of features based on time series trends not assessed by these methods.

In the current study we presented a machine learning model for the prediction of pAKI 3, together with its validation on the same population of the development cohort, and the external validation on the unseen population extracted from eICU and MIMIC-III, two large multi-center clinical databases.

## Materials

### Data sources

The data used in this study derived from the following sources:

- MIMIC-III [16], a single-center database from patients admitted at the Beth Israel Deaconess Medical Center in Boston (MA) from 2001 to 2012 (61,532 admissions, 46,476 distinct patients). In our exploration, we made use of the improvements introduced in MIMIC-III v1.4, released on September 2, 2016 [17]. The enhanced data quality and the considerable addition of diverse data elements provided valuable support for our analyses;
- eICU collaborative research multicenter database [18], a multicenter database which contains data from 208 different United States ICU wards, registered from 2014 to 2015 (200,859 admissions, 139,367 patients);
- AmsterdamUMC database (AmsterdamUMCdb) [19], a single-center database from patients admitted at the University hospital ICU of Amsterdam, in the Netherlands between 2003 and 2016, for a total of admissions (23,106 admissions, 20,109 distinct patients);
- MargheritaTre database, developed by the Group for the Evaluation of Interventions in Intensive Care Medicine (GiViTI) founded in 1991 [20] and available for research purposes. We were granted access to data collected from 2001 to 2022 in 27 different Italian centers (60,430 ICU admissions, 55,702 patients).

Unit of measure of these registries were horizontally standardized to build the present homogeneous cohort study.

The development cohort for this study was randomly drawn from the MIMIC-III and AmsterdamUMC databases for two key reasons: first, they represent older cohorts, allowing testing the generalizability of the prediction tool on newer cohorts. Secondly, they are both single-center databases, providing an opportunity to assess the performance of the model on a more diverse multicenter cohort, as represented by MargheritaTre and eICU databases. This approach ensures the evaluation of the model's effectiveness across different time periods and diverse healthcare settings.

### Persistent acute kidney injury stage 3 definition

While the definition of AKI and its staging has been formalized by the “*Kidney Disease: Improving Global Outcomes*” (KDIGO) organization during its 2012 annual conference [21], the definition of persistent AKI stage 3 has not been unanimously accepted. As primary prediction endpoint, we defined Persistent AKI Stage 3 (pAKI3) as a combination of AKI Stage 3, lasting at least 72 h (T72) or AKI Stage 3 lasting at least 48 h and leading to death (D48) or the initiation of renal replacement therapy after more than 24 h of AKI Stage 3, excluding cases where Stage 3 was solely attributed to RRT (RRT24). The rationale of such categorization, relies on the idea of selecting those patients presenting AKI 3 who were more likely to need renal replacement therapy. In Suppl. Info S1 Section the rationale for the choice of the endpoint is described.

We defined any portion of an ICU stay with constant AKI stage 3 a *severe AKI event.* If the severe AKI event is marked as persistent stage 3, we call it a *persistent event*. We use the notation T_p_ to indicate the time of the persistent event, i.e., the first hour of a persistent event.

We say that a patient underwent transient AKI if no persistent event occurred during his stay.

The baseline serum creatinine (bSCr) was established by modifying the nadir serum creatinine (sCr). For ICU stays with RRT, we chose the minimum sCr between admission and RRT initiation. For stays without RRT, the minimum sCr among all patient-related stays was selected, excluding those involving RRT.

Additional details on the motivation behind our pAKI3 definition can be found in Suppl. Info S1 Section. In Suppl. Info S4 Section we presented the labeling of time samples close to T_p_.

### Variables selection and uniform resampling

We started the analysis by considering a series of clinical, anamnestic, and demographic data, generally believed to have a role in AKI development (Suppl. Info S1 Table). This table also highlights the variables that have been measured in more than 70% of the stays of AmsterdamUMCdb, MIMIC-III and eICU. These are the variables used by our digital prediction model. The machine-learning model uses clinical variables measured during all AKI Stage 2 and AKI Stage 3 episodes in the ICU stay of the patient till the onset of the first Persistent AKI Stage 3 episode. After the first Persistent AKI Stage 3 event, subsequent AKI episodes were not analyzed by the model. The model also leverages time-series of clinical variables as described in Sect. 3.5 on Feature Engineering.

The two external validation cohorts take on two distinct roles: while eICU is a real-world example of how our model generalize in ICU with similar data quality and data availability, MargheritaTre will assess the robustness of the model under high rate of missing features, that could degrade model performances. As shown in S1 Table, Suppl. Info., the percentage of ICU stays having at least a measurement of several clinical variables used by the model was significantly lower.

Table S2 shows standardized vital signs and lab results; values beyond limits were flagged and excluded. Each variable has a specified validity duration (Max_gap). Urine output was transformed from volume to flow, considering the distance between measurements based on Max_gap. Hourly resampling was done within the ICU stay timeframe, enabling hourly AKI stage computation and prediction endpoint determination for serum creatinine and urine output.

### Inclusion and exclusion criteria

We included ICU adult (≥ 18 years of age) patients having recorded sex, height, and weight. To assess KDIGO staging, concomitant measurement of serum creatinine (sCr) and urine output (UO) starting in the first 12 h of the ICU stay were required. By looking at the last sCr measurement and the last 24 h of urine output we computed the AKI stage at any hour of any stay; then we selected only those stays having at least one hour marked as AKI stage 2 (for UO resampling technique, please refer to Suppl. Info. S2 Section).

Exclusion criteria can be divided into four categories: demographic exclusion, staging exclusion, imminence exclusion and aambiguity exclusion. A graphical version of the inclusion and exclusion criteria can be found in Table 1.

**Table 1:** Exclusion criteria

Type of exclusion	Exclusion
Demographic exclusion	Age < 18 years
	Height < 130 cm and > 200 cm
	Renal Transplant at admission
Staging exclusion	Less than 24 h of data
	Oliguria (no Anuria) at start of data
Imminence Exclusion	Death after less than 48 h of AKI stage 3
	RRT after less than 24 h of AKI stage 3
Ambiguity exclusion (subsequent events)	Primary: 1. AKI stage 3 for less than 72 h 2. No measurement till 72nd hour 3. Discharge
	Death: 1. AKI stage 3 for less than 48 h 2. No measurement till 48th hour 3. Death
	RRT 1. AKI stage 3 for less than 24 h 2. No measurement till 24th hour 3. RRT administration

Legend: n-th hour is meant from the first hour of the persistent event

Further details on inclusion and exclusion criteria can be found in Suppl. Info. S3 Section and its graphical version in Suppl. Info. S2 Figure.

### Model definition and feature engineering

For this study, we selected a gradient boosted trees approach. This model lends itself well to optimization for imbalanced data issues, as seen in the context of predicting pAKI3, which exhibits a low incidence rate. Furthermore, its output can be readily elucidated using SHAP values [22].

Selecting this technique also allowed to manage high dimensional data: at each timestamp, the model uses a vector of 542 components encapsulating statistical features of the near past of each variable, distance from the last measurement and whether the variable is missing.

The features were generated from medical variables by analyzing the time series, their differences, and macro measurements in the recent past. For instance, the feature "creatinine_diff_48h_mean" represents the mean value over the last 48 h in the time series, which is obtained by taking the differences between consecutive serum creatinine measurements.

For each medical variable, we included a time series indicating the duration since the last measurement and, excluding creatinine and diuresis, a boolean time series indicating the absence.

We trained and optimized the model on 70% of the ICU stays of AmsterdamUMCdb and MIMIC-III, while we calibrated the cutoff threshold and validated the model on the remaining 30%.

Additional details on problem formulation and sample labeling are described in Suppl. Info S4 Section.

### Model performance

To assess the performance of this model, we considered the computed risk scores only for those time samples that were associated to AKI stage 2 and 3. This means that, once the patient displays AKI 2, we want the model to predict whether he will develop persistent pAKI3 or, once the patient displays AKI 3, the model has to predict that this severe event will translate into a persistent event.

To penalize late alarms, stays displaying persistent stage 3 and transient AKI were treated differently: transient ICU stays are considered in their entirety, while the risk scores of a persistent ICU stay are computed up to timestamp ${T}_{p}+12$ hours.

For each ICU stay we took the maximum rate. For any given cutoff point the stay will be classified as predicted persistent if and only if its maximum rate exceeded the cutoff within 12 h from T_p_. We call this measurement strategy the MAX_Metric.

Even though the analysis of the Receiver Operating Characteristic (ROC) curve is the golden standard for assessing the performance of a clinical predictive models, in imbalanced problems the Precision Recall (PR) should be preferred [23]. The ROC curve, while useful, can be misleading in imbalanced datasets by resulting in high auROC values only due to the low incidence of the prediction endpoint. This is because ROC curve treats true positives and false positives equally. In situations with low incidence of prediction endpoints (rare conditions), and where model false positives may have serious consequences, precision-recall metrics are more suitable. Precision-recall analysis accurately evaluates positive predictions, emphasizing precision (Positive Predictive Value) and highlighting the impact of false positives. This is especially important in developing prediction tools for low-incidence conditions (such as persistent AKI Stage 3) and where misdiagnosis could lead to significant consequences for patient outcomes. We computed both curves for the MAX_Metric as well as their areas, i.e. auROC and auPR.

As cutoff criterium, we selected the threshold corresponding to 80% sensitivity on the internal validation cohort. For this cutoff we computed sensitivity, namely recall or true positive rate (TPR), specificity, namely 1-false positive rate (FPR), and precision, namely predictive positive value (PPV).

Models measured on dataset with low incidence display low precision [24]. We then computed the theoretical precision assuming prevalence of persistent AKI stage 3 of 10%.

Finally, for any persistent stay predicted as persistent we computed the distance from T_p_, i.e., the lead time of the alert.

To evaluate potential biases of our model across diverse patient sub-groups, we evaluated the Average Odds Difference metric from fairness analysis statistics. This metric evaluates how much the model is able accurately identify positive and negative cases in binary classification models, promoting consistent performance across various subgroups like race or gender. This principle, akin to medical tests, underscores the importance of fairness and reliability in healthcare by demanding consistent model performance across demographic or clinical subgroups. The computation of the Average Odds Difference (AOD) metric is as a single-metric approach to the Equalized Odds. Given a monitored sub-group, let the reminder of the cohort the reference group. The AOD of the monitored group is defined as:

$$AO{D}_{monitored}= \frac{\left(FP{R}_{monitored}-FP{R}_{reference}\right)+\left(TP{R}_{monitored}-TP{R}_{reference}\right)}{2}$$

Here, subscripts denote the subpopulation for which the metric is being calculated.

The ideal value of this metric is 0 and fairness for this metric is between −0.1 and 0.1[25]. All the analyses were both conducted on internal and external validation cohort with the fixed threshold described above. We evaluated the AOD according to age, comorbidities, reason for admission, ICU type and gender.

## Results

### Dataset preprocessing

The selection procedure described in section "Model performances" identified: serum creatinine, urine output, hematocrit, hemoglobin, bicarbonate, blood urea nitrogen, chloride, glucose, heart rate, platelets, potassium, sodium, white blood cells count.

**Fig. 1:**
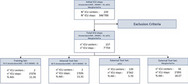
Data splitting

As shown in and Fig. 1, starting from a total of 345,927 ICU admissions we obtained: 1607 stays and 11.2% incidence in the AmsterdamUMCdb, 1497 stays and 11.4% incidence in the MIMIC-III, 3562 stays and 5.7% incidence in the eICU dataset coming from 139 centers, and 1093 stays and 18.6% incidence in the MargheritaTre dataset from 16 centers.

Among those ICU stays that displayed AKI stage greater than 1, subsequent exclusions are described in Suppl. Info S2 Section and Suppl. Info S5 Table. In Suppl. Info S6 Table we show the percentages of the four final classes defined in section "Hyperparameter tuning and feature importance": Transient AKI, T72, D48 and RRT24.

The 88% of the AmsterdamUMCdb cohort has transient AKI, 8.15% has persistent AKI stage 3 due to primary endpoint T72, 2.99% has persistent AKI stage 3 due to RRT administration, while 0.06% is persistent due to death after prologued AKI stage 3. In MIMIC-III 88.58% of stays displayed transient AKI, in 8.82% of stays the patient develops persistent AKI stage 3 due to T72 endpoint, 2% was administered with RRT after AKI 3, and 0.60% died after AKI 3.

In the external eICU and MargheritaTre validation cohorts we find 94.30% and 81.43% Transient AKI, 4.21% and 16.47% T72 endpoint, 1.12% and 0.37% RRT24 endpoint, and 0.36% and 1.74% D48 endpoint respectively.

These final populations are described in detail in Suppl. Info S7 Table according to sex, age, ethnicity, reason for admission, comorbidities, and ICU characteristics.

### Hyperparameter tuning and feature importance

**Fig. 2:**
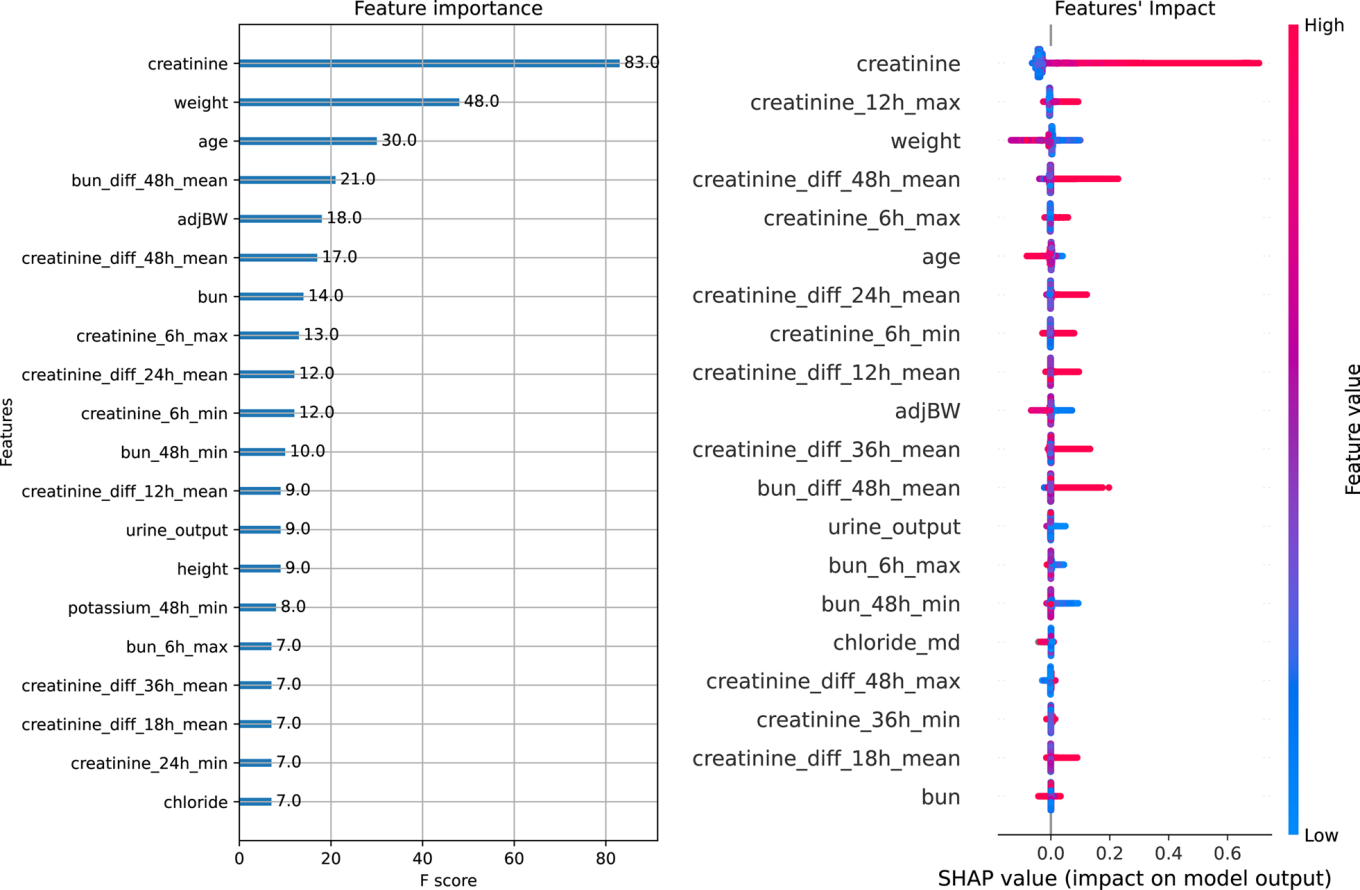
Feature Importance of the first twenty features in term of F score and SHAP value

Thanks to the high L1 regularization parameter, out of the 542 features only 214 have a non-null feature importance. In Fig. 2 we showed how sCr and its derivatives are the most important features, still other features can contribute to the prediction task.

### Model performances

In Table 2 we presented the performances of the final predictive model using the MAX_Metric.

**Table 2:** Models’ results, MAX_Metric on validation cohort

Dataset	ICU Stays	Persistent AKI Incidence (%)	auROC	auROC IQR	auPR	auPR IQR
Final model						
AmsterdamUMCdb	531	11.11	90.42	[87.57, 93.69]	48.05	[33.40, 58.94]
eICU	3562	5.70	93.58	[92.37, 94.85]	44.57	[36.90, 51.07]
MIMIC-III	495	11.52	92.63	[90.33, 95.50]	54.03	[40.14, 66.29]
MargheritaTre	1093	18.57	85.29	[83.17, 87.54]	49.78	[42.82, 56.29]

In the internal AmsterdamUMCdb and MIMIC-III validation cohort the model achieved auROC 0.90 and 0.93 respectively, while it reached auPR 0.48 and 0.54. In the external eICU and MargheritaTre the model achieved auROC 0.94 and 0.85 respectively, with auPR of 0.45 and 0.50.

In Table 3 we reported the performance of the models for the threshold that better represent an 80% sensitivity on the combined AmsterdamUMCdb and MIMIC-II internal validation cohort. For these two datasets, this choice achieved specificity of 0.83 and 0.86, and Precision (Positive Predictive Value) of 0.38 and 0.42 respectively. For the eICU dataset the same working point corresponds to 0.93 sensitivity, 0.82 specificity and 0.24 precision. For MargheritaTre we achieve 0.73 sensitivity, 0.78 specificity and 0.43 precision. When evaluated on the same hypothetical incidence of 10%, Precision is 0.37 in eICU, while on MargheritaTre it is 0.27.

**Table 3:** Performance for fixed 80% sensitivity MAX_Metric on validation cohort

Threshold	Dataset	F1	Sensitivity	Specificity	PPV	NPV	PPV (10% prevalence)
**Final model**							
0.36435273	AmsterdamUMCdb	51.85	81.36	83.47	38.1	97.28	35.36
	eICU	37.98	92.61	82.47	24.2	99.46	36.99
	MIMIC-III	54.66	77.19	86.3	42.31	96.67	38.50
	MargheritaTre	54.25	73.40	77.87	43.02	92.77	26.93

**Fig. 3:**
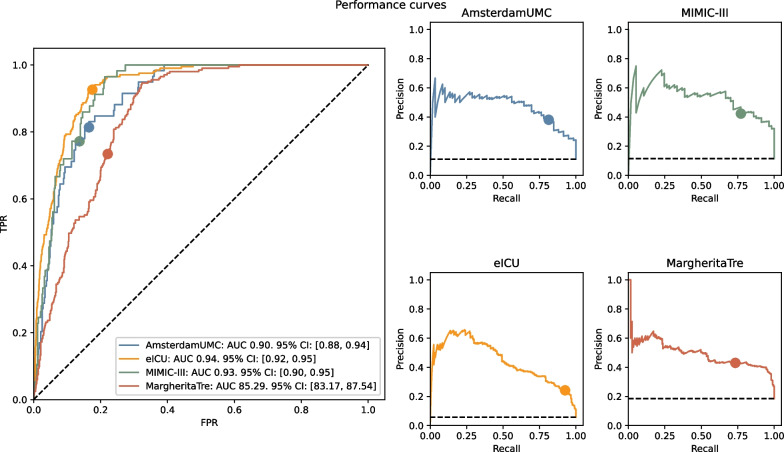
ROC and PR curves of final predictive model on different datasets (MAX_Metric). Dashed line is the random classifier

Figure 3 displays the ROC and PR curves of the model across four validation sets. Additionally, the performance at a fixed threshold is highlighted by a round marker. Dashed black lines represent the performances of the theoretical random classifier: the ROC corresponds to the bisector of the quadrant, and the PR curves are represented by horizontal lines with y-coordinates equivalent to the incidence of each dataset.

To evaluate the impact of using multiple variables for the prediction of pAKI 3, we also trained and validated baseline predictive models that leveraged only sCr as input feature. As shown in Suppl. Info. S11 Table and Suppl. Info. S12 Table, a model using the lone creatinine as biomarker achieved 0.91 auROC in eICU and 0.83 in MargheritaTre, while its auPR was 0.29 and 0.45 respectively.

### Model performances in different ICU subpopulation

We now explore the sub-populations where our model was unfair according to the Average Odds Difference (AOD) metric, that evaluates if the model performs with the same accuracy over different sub-populations with different characteristics. Optimal values of AODs are in the range [-0.1, 0.1].

In the AmsterdamUMCdb internal validation cohort (Suppl. Info S9 Figure) the AOD is outside the optimal [-0.1, 0.1] range for patients admitted for Trauma: AOD_Trauma_ = -0.23. This subpopulation represents 4% of the patients, of which only 2 patients underwent a pAKI3 episode.

In MIMIC-III internal validation cohort (Suppl. Info S11 Figure), we AOD_Sepsis_ = −0.27 and AOD_Cancer_ = -0.38. Patients with cancer do not display evident bias in the population and further analysis should be conducted. Septic patients represent only 5% of the population. $AO{D}_{age\in [{18,39}]}$ = 0.21, this group represents only 3% of the population and the persistence incidence is 18%, significantly higher than the 11% incidence encountered in the whole dataset.

In the external eICU validation cohort (Suppl. Info S10 Figure) we encountered AOD_Asian_ = −0.40. Asian population represents only the 0.56% of the dataset. Moreover, only 2% of these 20 individuals were positive, a small incidence if compared with the 5.7% incidence of the whole dataset. In the same cohort AOD_CKD_ = 0.14.

In MargheritaTre (Suppl. Info S12 Figure) AOD_CKD_ = 0.19.

## Discussion

We have shown that routinely acquired data, analyzed with a novel ML model, can predict the onset of pAKI 3 in a large international and multi-centric cohort of intensive care patients. Given our analysis on external validation datasets, we can assert that our algorithm generalizes well on external eICU validation cohort, and fairly in MargheritaTre, which is characterized by higher percentage of missing data, as shown in Table S1, Suppl. Info (with a 0.09 difference in AUC across validation sets). The comparison of the performance of our model with that of sCr as biomarker shows that our model is an improvement in clinical practice.

Pattern recognition on medical variables can become a powerful tool in clinical management of patients with AKI in the ICU. In addition, using explainable models such as trees can potentially advance the understanding of renal mechanisms.

Our studies can be directly translated to a data pipeline which uses EHR collected in ICU for the continuous prediction of future pAKI 3. The resulting risk score would aid in the prompt determination of which patients would require an early-start of RRT and which patients are likely to recover. To assess the cutoff criterium we used only the internal AmsterdamUMCdb and MIMIC-III cohorts and applied the threshold to the external eICU and MargheritaTre cohorts; hence, no calibration on unseen population was employed. This means that a prediction tool built upon the model that we developed can perform excellently from day zero.

In the present study the model which was chosen for early prediction of pAKI3 is a boosted tree. There are multiple reasons why this model is preferable to more complex models such as artificial neural networks (NN).

Firstly, the model can easily handle missing data, learning the optimal value to use for imputation through the analysis of the optimal path in the tree. This reflects in robustness under missing data in the MargheritaTre cohort.

Secondly, the model manifests outstanding performance. In recent years NN have been employed to predict AKI development [26, 27]. Still, from a systematic review on the topic, boosted trees showed the best performances in predicting AKI [28].

Lastly, NN are often built as a black box, the correlation between variables and the produced risk scores are unclear, hence clinicians are sceptical in using managerial tool which produce no explanation. Simple linear regression is still used nowadays to ensure interpretability. With our tree approach we preserve understanding of the model, while outperforming linear regression.

Furthermore in previous study [14, 26, [27] the related models have been trained and evaluated only on the MIMIC-III, while we used horizontal integration to show that our model can generalize on external datasets and unseen populations. In recent years, generalizability of predictive models has been shown to be a critical issue in real-world implementation of these tools [29].

Works that applied ML to the prediction of persistent AKI stage 3 used the definition of AKI stage 3 for 48 h. This condition has a much higher incidence than the 72 h AKI stage 3 that we selected.

In a previous research, a total of 5984 septic patients with AKI were selected from MIMIC-III, 3805 (63.6%) of whom developed persistent AKI stage 3 [14]. The selected model achieved auROC 0.76 (95% CI 0.74–0.78) on the internal validation cohort. Compared to our model this work focused only on septic patients, achieved lower performance, and was not validated on any external cohort.

Jiang et al. enrolled 955 patients admitted to the ICU of Dongyang People’s Hospital after surgery complicated by AKI, and persistent AKI stage 3 incidence was 39.4% [15]. The model was externally validated on 3170 patients of MIMIC-III who were first admitted to the ICU and underwent surgical treatment; persistent AKI stage 3 incidence was 45.1%. The selected model exhibited auROC 0.69 on the external validation cohort. Compared to our work, this model was externally validated only on a single-center ICU registry, while we performed external validation on two multi-center medical registries and on a much more variable cohort of patients.

This study has several limitations. Our analysis is retrospective, indicating the need for a prospective study to validate the model's performance. Additionally, we require a prospective interventional study to evaluate the impact of our AKI risk prediction score on clinical outcomes and how it can be applied. A prospective observational study to confirm our model's performance in real-world scenarios is currently underway.

While the random selection of the development cohort from MIMIC-III and AmsterdamUMC databases has advantages, it introduces potential limitations. Using older cohorts may not fully capture evolving healthcare trends, and focusing on single-center databases may limit the model's exposure to multicenter complexities. These considerations should be acknowledged when interpreting the study's findings.

The second limitation is linked to the definition of AKI as KDIGO guidelines. The computation of the staging requires a baseline for sCr which has no formal definition for retrospective studies. In our analysis we used the nadir (minimum) in-hospital value when a pre-hospital baseline was not available. This approach was found to achieve 81.7% sensitivity and a specificity of 79.8% for the diagnosis of AKI, when compared with actual pre-hospital admission baseline values, and only 2.8% misclassified KDIGO stage 2 or 3 [30, 31]. Other techniques for bSCr imputation can be explored.

Third limitation is the impossibility of discriminating between AKI types (nephrotoxic, inflammatory, versus septic). This limitation also affects most prospective studies [11]. Multi-class classification could solve the problem of early detection of AKI types.

Moreover, as highlighted by the AOD metrics results, and in particular AOD_CKD_ measured on the external validation cohorts, subsequent studies should focus on CKD patients (excluding patients with CKD or using CKD as input to the model for performance and generalizability improvement) and improving model performances on sepsis, cancer and Asian patients by including more patients with these condition in future patient cohorts. Low prevalence of the aforementioned subclasses in the training dataset of the model may have had a negative effect on the model ability to perform accurately on those patients. Moreover, a low number of patients with these characteristics in the validation cohorts make the AOD metric calculation less reliable.

## Conclusions

Through retrospective analysis of extensive ICU data, this study highlights the potential of AI, specifically a boosted tree model, in predicting the risk of persistent AKI stage 3. Our model can predict elevated Persistent AKI Stage 3 risk in advance using routine EHR data. While retrospective analysis has limitations, our multi-center study, outperforming existing models, suggests real-world implementation for proactive AKI management.

### Supplementary Information

Additional file S1 Primary Endpoint definition. Additional file S2 Uniform resampling. Additional file S3 Exclusion criteria. Additional file S4 Problem formulation and sample labeling. Additional file S5 Example of risk score.

## Data Availability

Deidentified patient data from the Medical Information Mart for Intensive Care III (MIMIC-III) v1.3 database is publicly available at https://mimic.physionet.org/. Deidentified patient data from the AmsterdamUMC database (AmsterdamUMCdb) is publicly available at https://amsterdammedicaldatascience.nl/#AmsterdamUMCdbdb. Deidentified patient data from the eICU Collaborative Research Database (eICU) is publicly available at https://eicu-crd.mit.edu/. The MargheritaTre dataset is available upon request as there are ethical restrictions on sharing data publicly and the data is owned by a third-party organization. Agreement on data sharing must be requested to Istituto di Ricerche Farmacologico Mario Negri and evaluated by an ethical committee.

## Abbreviations

ADQI: Acute Disease Quality Initiative
AI: Artificial Intelligence
AKI: Acute Kidney Injury
AOD: Average Odds Difference
auPR: Area under the Precision-Recall curve
auROC: Area under the Receiver Operating Characteristics
bSCr: Baseline Serum Creatinine
FPR: False Positive Rate
GiViTI: Group for the Evaluation of Interventions in Intensive Care Medicine
ICU: Intensive Care Unit
KDIGO: Kidney Disease: Improving Global Outcomes
MIMIC-II: Medical Information Mart for Intensive Care III
ML: Machine Learning
PPV: Predictive Positive Value
PR: Precision-Recall
ROC: Receiver Operating Characteristic
sCr: Serum Creatinine
T_p_: Time of Persistent event, i.e., first offset of a persistent event
TPR: True Positive Rate
UO: Urine Output
