# Supplementary material for: Development and external validation of a machine learning model for the prediction of persistent acute kidney injury stage 3 in multi-centric, multi-national intensive care cohorts
Source: Crit Care. 2024 Jun 4;28:189. doi: 10.1186/s13054-024-04954-8 (PMC11149298; doi:10.1186/s13054-024-04954-8)
Supplement: Supplementary file 1 — Additional file S1 Primary Endpoint definition. Additional file S2 Uniform resampling. Additional file S3 Exclusion criteria. Additional file S4 Problem formulation and sample labeling. Additional file S5 Example of risk score. [file 13054_2024_4954_MOESM1_ESM.docx]

# **Supplementary**

# Primary Endpoint definition

While the definition of Acute kidney injury (AKI) and its staging has been formalized by the KDIGO organization during its 2012 annual conference [1], the definition of persistent AKI has not been unanimously accepted. We found two main definitions for Persistent-AKI:

- **(T48)**: A Persistent AKI is an AKI stage 3 retained for at least 48 hours [2]
- **(T72)**: A Persistent AKI is an AKI stage 3 retained for at least 72 hours [3]

We chose the latter for multiple reasons. Firstly, the definition **(T72)** is consistent with long term effect of AKI persistence (figure 3 in [4]). Secondly, requiring the patient to persist in AKI 3 for a longer time is a stricter constraint: the incidence is smaller. In the context of Machine Learning this means that the problem of classifying persistent events is highly imbalanced. Whole books have been devoted to the problem of performance degradation connected to imbalanced datasets [5], [6]. Choosing the latter definition means choosing a harder research path. Finally, we want to compare our statistical algorithm with current state-of-the-art research of biomarkers for nephrology [7], [8] which use the Kellum’s definition.

Since we want to measure the impact of prolonged AKI 3, we also consider the following endpoints in the definition of Persistent-AKI

- **(D48)**: AKI stage 3 for at least 48 hours, but less than 72, then death
- **(RRT24)**: AKI stage 3 for at least 24 hours, but less than 72, then RRT initiation

We consider the following definition as Persistent AKI Stage 3, the Primary Prediction endpoint of the study:

“A patient is said to display Persistent AKI Stage 3 if one of the conditions:

**(T72)**, **(D48)** or **(RRT24)**

manifests”

# Uniform resampling

Medical signs and measurement are characterized by a 3-tuple identifying the unique ICUstay_ID, the unique chart_time and the unique sign_ID, identifier of the biological measurement in the analyzed databases. Timestamps are not uniformly distributed, and each selected medical sign has its own acquisition rate as in Suppl. Info. S3 Table.
We show in Suppl. Info. S1 Algorithm the data pipeline that transforms each sequence of measurements into a uniformly sampled time series.

A special treatment is applied to the urine output in Suppl. Info. S2 Algorithm. First, measurements are transformed from volume to rate in ml/h. Then the resampling technique works as follow:
Consider a patient measured with 50 ml/h at 9:40am, and 60 ml/h at 10:10am and no other measurements till 11:00am. In the interval 10:00am -11:00am the first rate is valid for the first 10 minutes and the latter is valid for the remaining 50 minutes. We assign

$$urine\_output(11:00am)=\frac{10m*50\frac{\mathrm{ml}}{h}+ 50m*60\frac{\mathrm{ml}}{h}}{60m}=58.3\frac{\mathrm{ml}}{h}$$

# Exclusion Criteria

## Demographic exclusion

Firstly, one of the exclusion criteria was the availability of patient demographic info. Urine output values were normalized by the adjusted body weight of the patient (adjBW).

AKI criteria, as KDIGO classification, require the baseline value of sCr (bSCr). Various methods for determining the baseline value of sCr values have been described and there is not a universally accepted single method[9]. Due to the absence of pre-ICU admission Serum Creatinine measurements for most ICU patients, we used the nadir (minimum) in-hospital value of Serum Creatinine as bSCr. The existence of at least one creatinine measurement was another requirement. For patients being administered with RRT, we selected the first available value of sCr as its baseline.

Once these demographic details are present, we also ask patients’ age to be greater than 18 years, their height h to be 130cm ≤ h ≤ 200 cm.

We excluded patient who underwent a kidney transplant before ICU admission.

## Staging exclusion

The aforementioned filters were accompanied by Persistent AKI Stage 3 specific exclusion criteria.

After having implemented a staging algorithm as the KDIGO guidelines (Table 2 in [1]) we applied it to the resampled time series of urine output and sCr.

The two aligned time series are characterized by a first and a last offset that do not necessarily correspond to ICU admission and discharge time. Patients having less than 24 hours of aligned urine output and sCr were excluded.

Once the KDIGO staging was computed, we retrospectively excluded patients that never reached AKI stage 2 or 3 during their ICU stay.

## Imminence Exclusion

In the definition **(D48)** and **(RRT24)** imposed a temporal constraint on Persistence AKI Stage 3 defined by RRT and death events. We did this to discriminate between patients whose prolonged AKI condition led to these events by patient who displayed imminent need of RRT or imminent risk of death.

For this reason, we excluded patients who initiated RRT after less than 24 hours of consecutive AKI 3 and patients who died after less than 48 hours of consecutive AKI 3

The first exclusion also considers the case in which the first AKI 3 staging was triggered by RRT administration.

## Ambiguity exclusion

As pointed out in Section 3.2 the sCr and normalized urine output time series span a time interval that do not necessarily overlap the whole ICU stay. This introduces ambiguity in the AKI and Persistent AKI Stage 3 staging.

One of the KDIGO definitions of AKI 3 staging is anuria for at least 12 hours. For a patient, we may have no measurement after ICU admission, then anuria. If the sum of these two intervals exceeded 12 hours, we excluded the patient due to staging ambiguity. The following exclusion criteria are similar.

We may have constant AKI 3 staging for less than 72 hours, then an interval of no measurement before ICU discharge. If the sum of these two intervals exceeded 72 hours, if the patient died inside the latter interval or if the patient initiated RRT inside the latter interval, we exclude the patient.

# Problem formulation and sample labeling

The goal of this study is the prediction of the persistence of a patient in AKI 3 as defined in Section **Error! Reference source not found.**. From a machine learning perspective, the prediction of Persistent AKI Stage 3 onset is finding patterns in the collected medical signs that may indicate the transition of a patient to a different state: the persistent state. As described in Section S2 we have availability of resampled measurements at each hour. We are interested only in the portion of ICU stay when it is possible to define creatinine and urine output. At each selected timestamp we would like to determine a transition probability.

We consider the following labeling of hourly samples of any patient:

- Let $[T_{p}, T_{d}]$ the interval of a persistent event:
  - $[T_{p}-24, T_{d}]$ is marked with 1.
  - Hours after T_d_ are excluded from the regression problem.
- Let $[T_{e}, T_{last}]$ a severe AKI event lasting more than 24 hours which is not also a persistent event, i.e., T_last_ -T_e_ < 72hours and T_last_ does not correspond with RRT administration or death offset:
  - Samples of $[T_{p}-24, T_{last}]$ that were not previously marked as 1 are marked with ½.
- Every other hourly sample is marked with 0

A graphical version of samples’ labeling is shown in Suppl. Info S1 Figure.

# Example of risk score

In the present document we have reported in S12 Figure and S13 Figure we plotted two examples of the computed risk rate of a patient of developing Persistent-AKI Stage 3. In S12 Figure we considered a patient that developed Persistent AKI Stage 3. The risk rate jumps above the selected threshold at the 52^nd^ hour of the ICU stay, while the first AKI stage 3 will occur at the 66^th^ hour. In S13 Figure we considered a patient that didn’t develop Persistent-AKI Stage 3 since its AKI 3 staging lasted for 60 hours (50^th^ to 109^th^ hour), then AKI went back to stage 2 for 23 hours, then 24 hours of AKI 3 (133^rd^ to 156^th^ hour), finally the patient went back to stage 2 and was discharged at the 219^th^ hour. The model recognized that the kidney injury was transient, hence the risk rate stayed below the selected threshold.

# Bibliography

[1] J. A. Kellum *et al.*, “Kidney disease: improving global outcomes (KDIGO) acute kidney injury work group. KDIGO clinical practice guideline for acute kidney injury,” *Kidney Int Suppl (2011)*, vol. 2, pp. 1–138, 2012.

[2] L. S. Chawla *et al.*, “Acute kidney disease and renal recovery: consensus report of the Acute Disease Quality Initiative (ADQI) 16 Workgroup,” *Nat Rev Nephrol*, vol. 13, pp. 241–257, 2017.

[3] J. A. Kellum, “Persistent acute kidney injury,” *Crit Care Med*, vol. 43, p. 1785, 2015.

[4] J. A. Kellum, F. E. Sileanu, R. Murugan, N. Lucko, A. D. Shaw, and G. Clermont, “Classifying AKI by urine output versus serum creatinine level,” *Journal of the American Society of Nephrology*, vol. 26, pp. 2231–2238, 2015.

[5] Y. Ma and H. He, *Imbalanced learning: foundations, algorithms, and applications*. John Wiley & Sons, 2013.

[6] A. Fernández, S. Garcı́a, M. Galar, R. C. Prati, B. Krawczyk, and F. Herrera, *Learning from imbalanced data sets*, vol. 10. Springer, 2018.

[7] S. M. Bagshaw *et al.*, “External validation of urinary C–C motif chemokine ligand 14 (CCL14) for prediction of persistent acute kidney injury,” *Crit Care*, vol. 25, pp. 1–8, 2021.

[8] E. Hoste *et al.*, “Identification and validation of biomarkers of persistent acute kidney injury: the RUBY study,” *Intensive Care Med*, vol. 46, pp. 943–953, 2020.

[9] E. D. Siew *et al.*, “Commonly used surrogates for baseline renal function affect the classification and prognosis of acute kidney injury,” *Kidney Int*, vol. 77, no. 6, pp. 536–542, 2010.

# Additional figures

## Sample Labeling


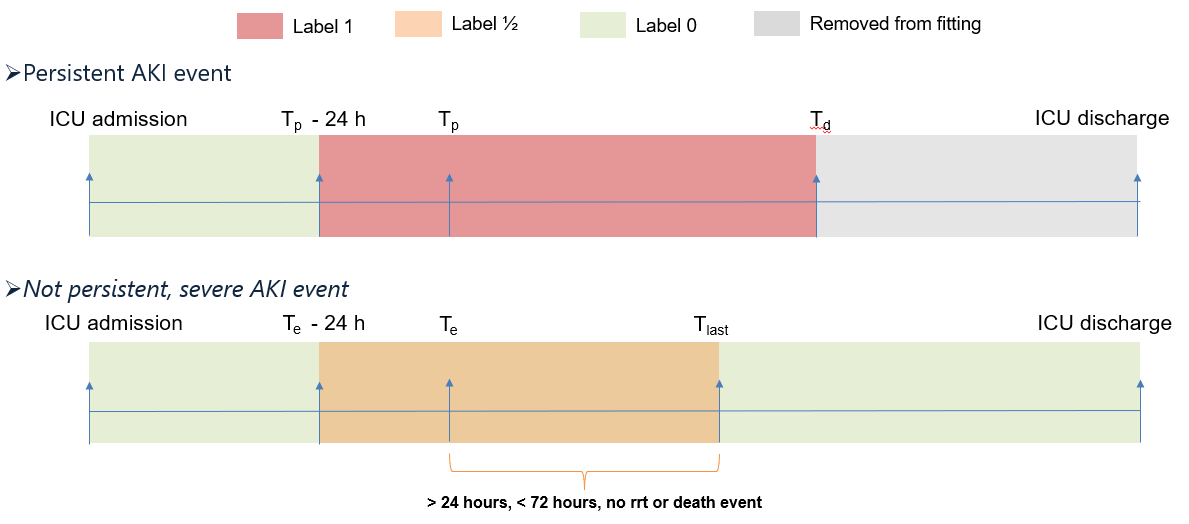


S1 Figure: Labeling of patients undergoing Persistent and Transient AKI


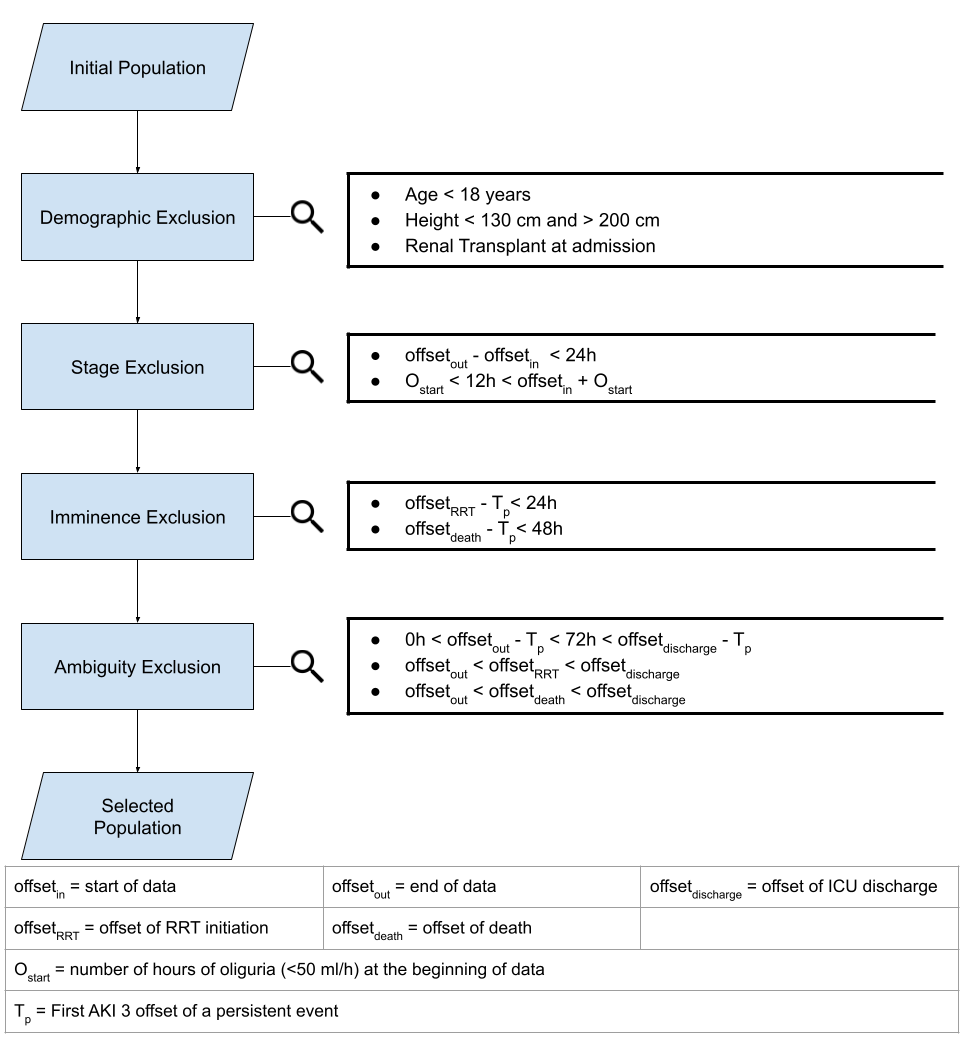


S2 Figure: Exclusion criteria: All time values are expressed in hours from ICU admission.


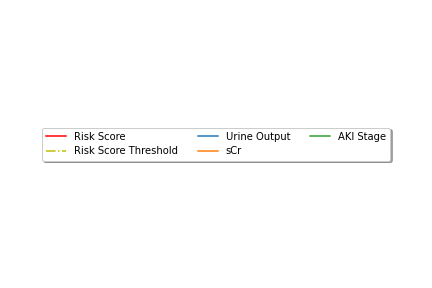

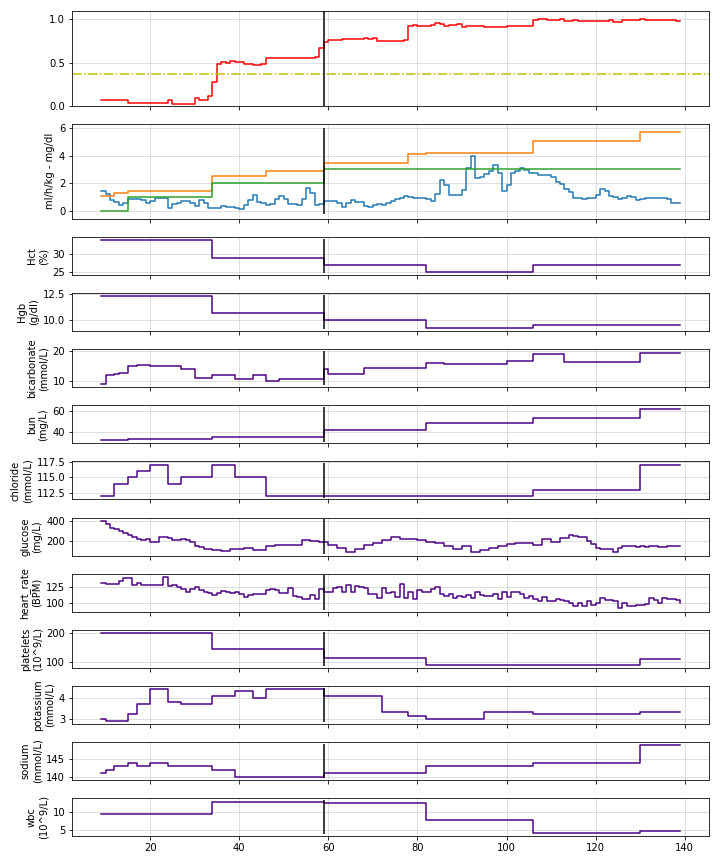


Figure 1 Example of an external test set patient (eICU dataset) with Persistent AKI. The vertical black line is T_p_.


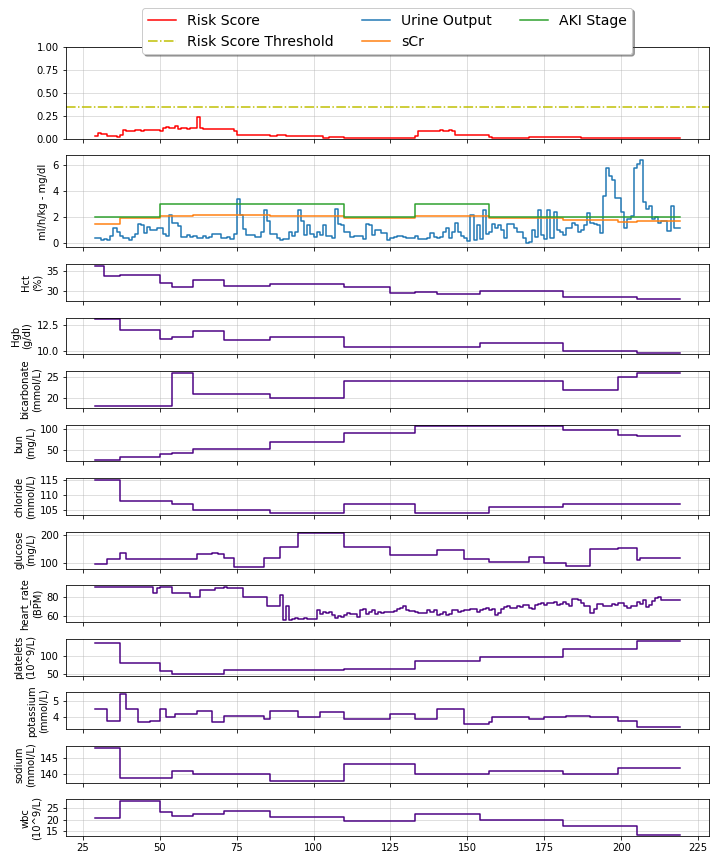


S8 Figure: Examples of model working on a test negative patient.

## Bias Detection


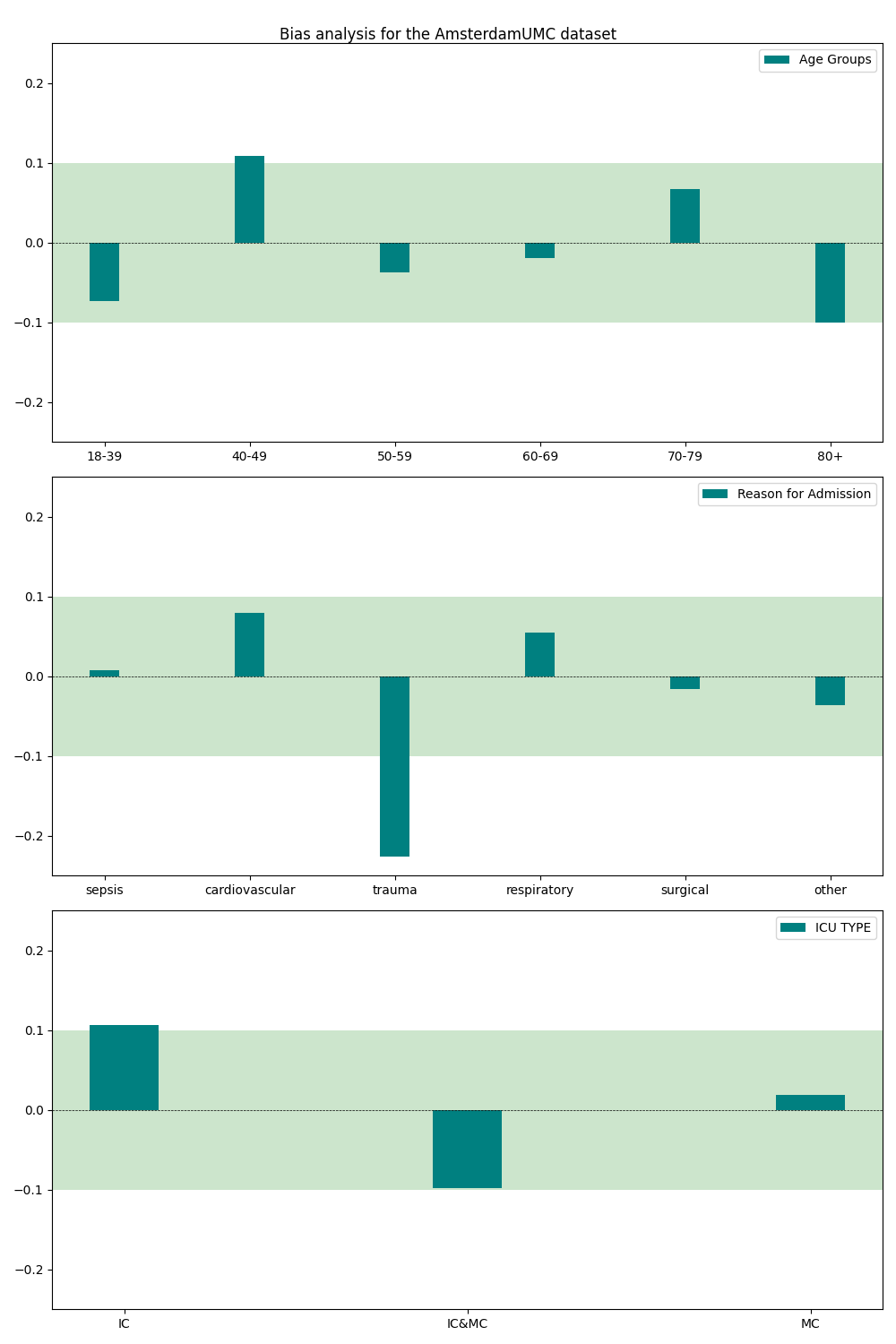


S9 Figure: Average Odds Differences for the AmsterdaUMC validation cohort


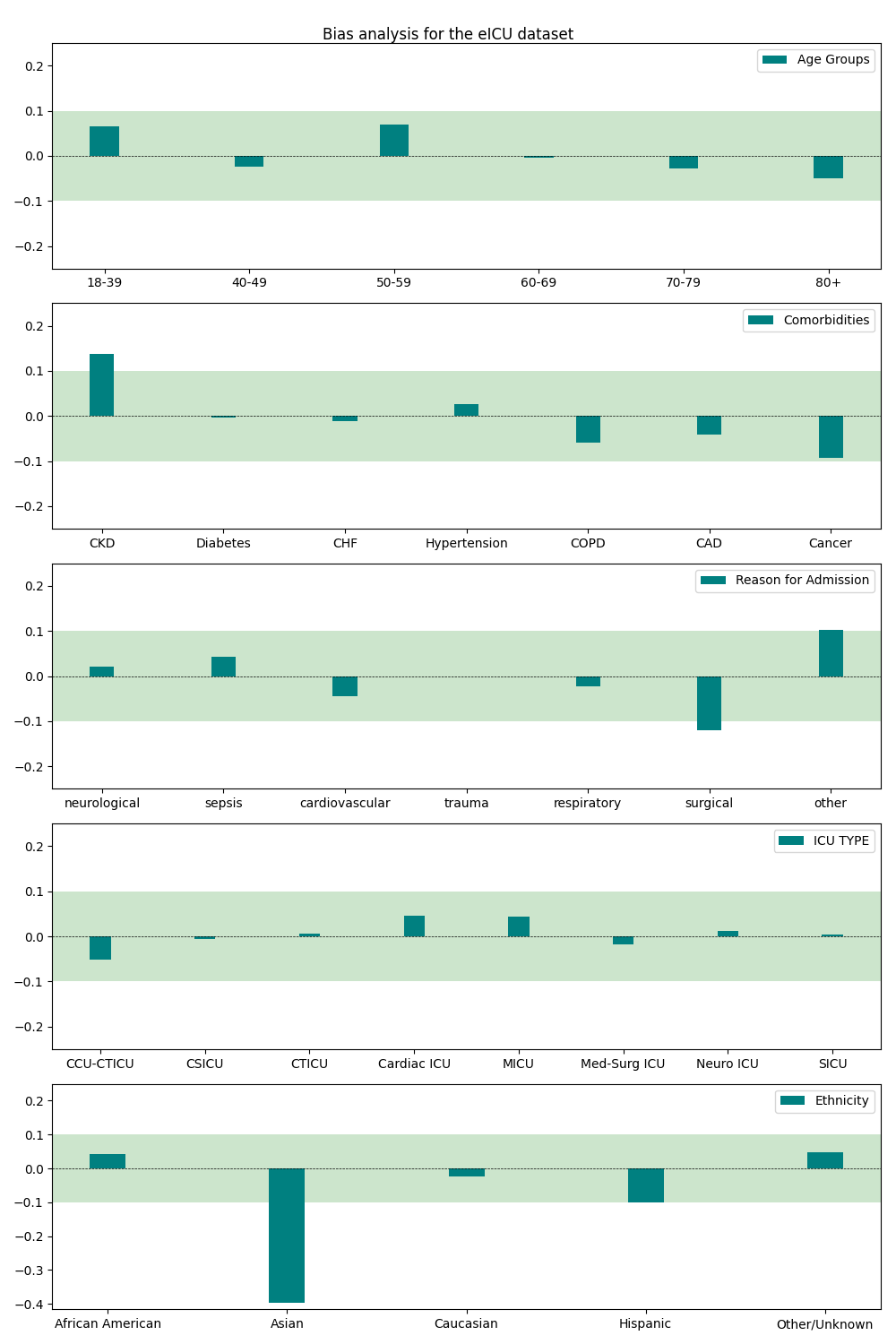


S10 Figure: Average Odds Differences for the eICU validation cohort


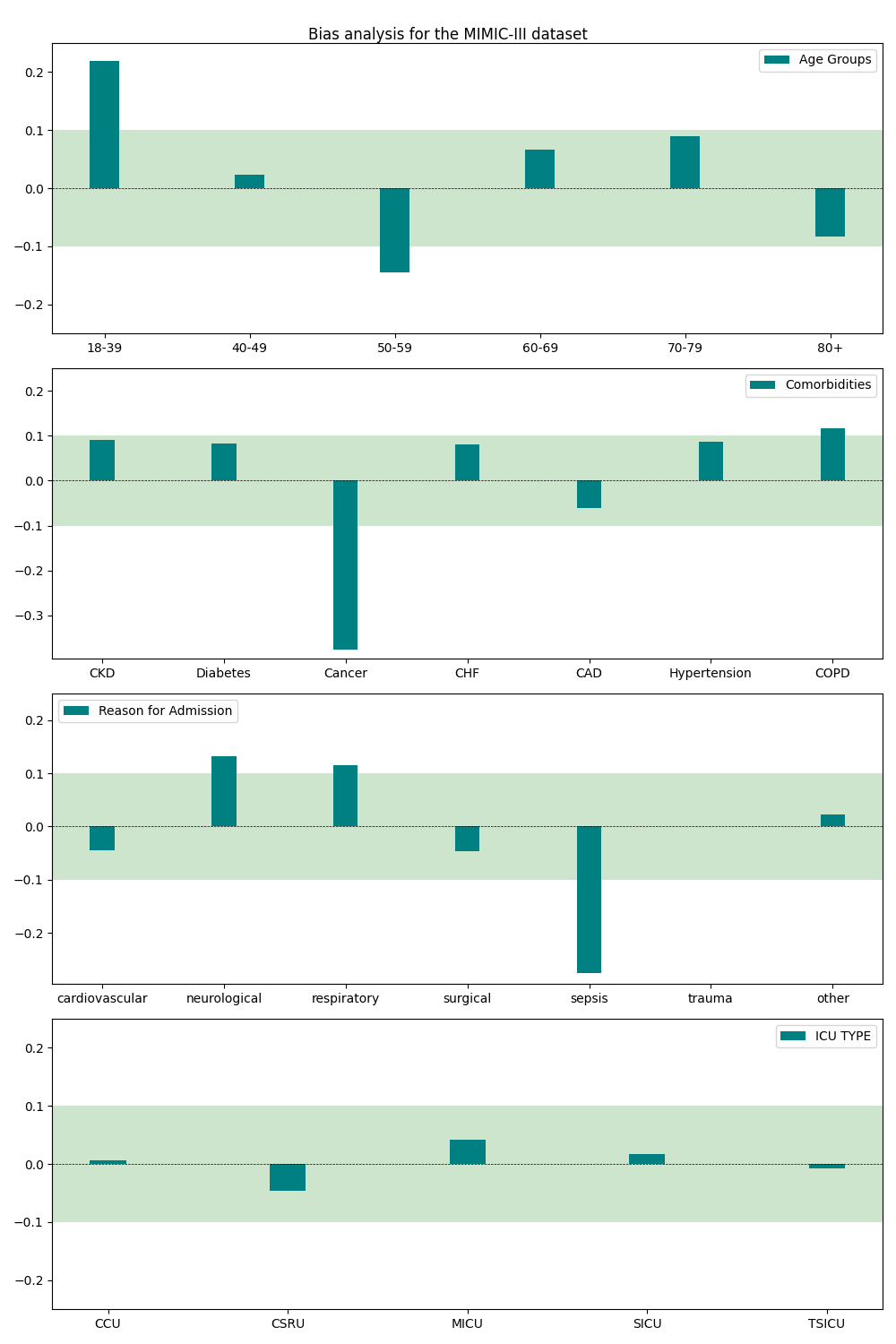


S11 Figure: Average Odds Differences for the MIMIC-III validation cohort.


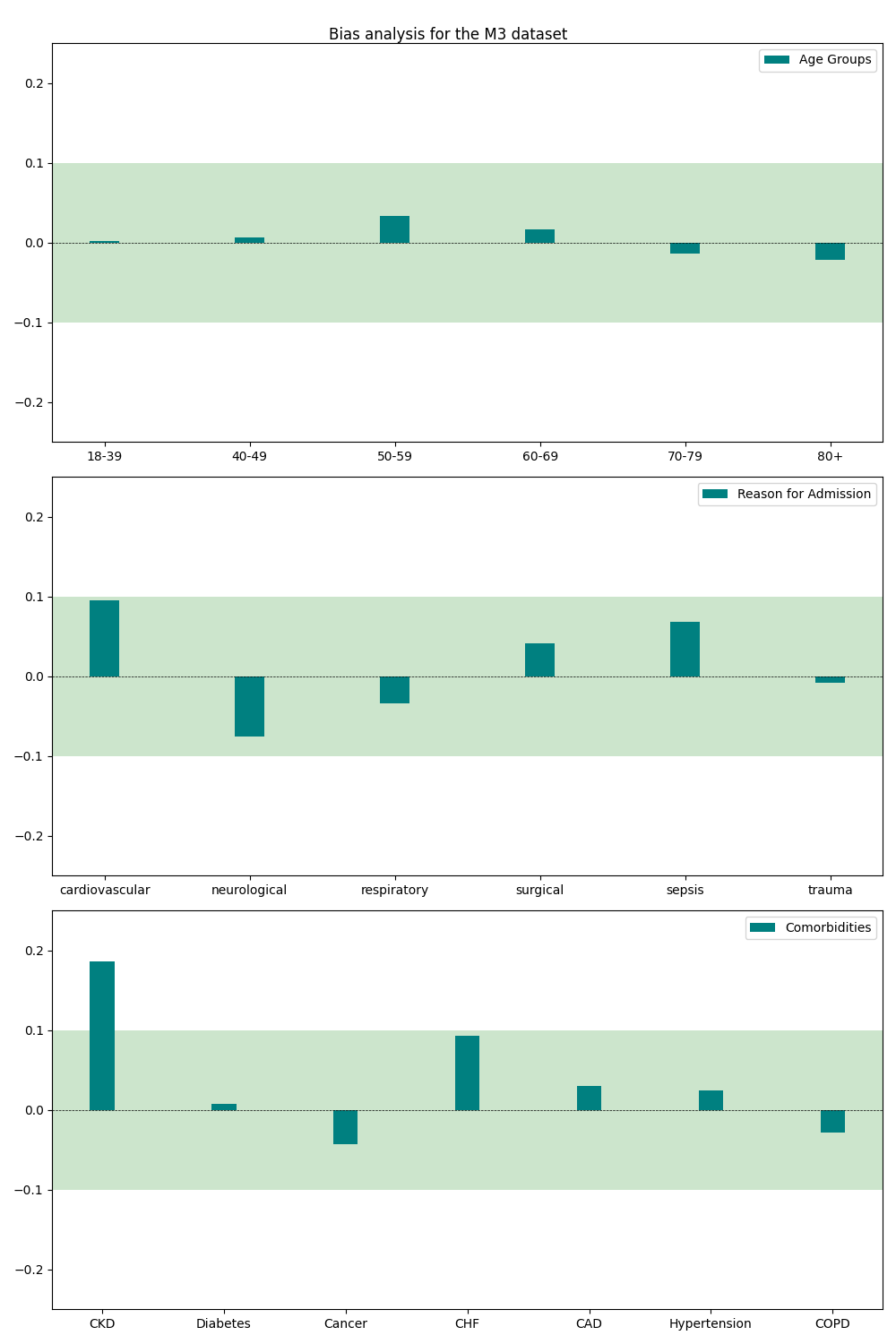


S12 Figure: Average Odds Differences for the MargheritaTre validation cohort

# Additional Tables

## Medical Signs analysis

S1 Table: Percentage of ICU stays having at least a measurement of the related medical sign.

|  | AmsterdamUMC | MIMIC-III | eICU | MargheritaTre |
| --- | --- | --- | --- | --- |
| Albumin | 80.74% | 53.37% | 71.22% | 14.81% |
| Anion gap | 74.93% | 90.39% | 75.56% | 0.00% |
| Bicarbonate | 97.07% | 90.58% | 91.86% | 15.28% |
| BUN | 76.58% | 87.41% | 94.67% | 20.19% |
| Chloride | 82.48% | 90.68% | 94.32% | 16.03% |
| CVP | 64.49% | 30.58% | 10.11% | 29.75% |
| DIASTOLIC | 44.10% | 34.77% | 21.73% | 95.75% |
| Eosinophils | 34.21% | 74.48% | 70.29% | 20.64% |
| FiO2 | 6.60% | 33.20% | 43.10% | 84.59% |
| Glucose | 99.24% | 86.83% | 95.97% | 16.45% |
| HTC | 96.43% | 97.47% | 94.47% | 25.18% |
| Heart Rate | 99.99% | 97.81% | 91.88% | 98.32% |
| HGB | 96.49% | 97.27% | 94.07% | 25.19% |
| Urine Output | 98.21% | 87.50% | 72.50% | 93.34% |
| Neutrophils | 30.59% | 74.48% | 65.32% | 21.08% |
| PaO2 | 96.88% | 29.96% | 42.55% |  |
| Platelets | 96.17% | 97.23% | 93.81% | 25.22% |
| Potassium | 94.61% | 90.74% | 94.66% | 22.79% |
| SaO2 | 99.99% | 67.33% | 82.70% | 77.34% |
| Serum Creatinine | 97.15% | 86.16% | 89.78% | 24.97% |
| Sodium | 94.75% | 90.72% | 94.78% | 22.81% |
| SYSTOLIC | 44.10% | 36.05% | 21.74% | 95.74% |
| Total protein | 8% | 5.71% | 68.18% | 12.43% |
| WBC | 96.17% | 97.25% | 94.15% | 25.32% |

S2 Table: Admissible values for the selected medical signs

| **Medical Sign** | UOM | Max_gap (h) | Lower bound | Upper Bound |
| --- | --- | --- | --- | --- |
| **Serum Creatinine** | **mg/dL** | 96 | 0 | 74 |
| **Urine output** | **ml/h** | 4 | 0 | 1000 |
| **HTC** | **%** | 96 | 0 | 100 |
| **HGB** | **g/dL** | 96 | 2 | 20 |
| **bicarbonate** | **mmol/L** | 96 | 0 | 40 |
| **BUN** | **mg/dL** | 96 | 0 | 200 |
| **chloride** | **mmol/L** | 96 | 60 | 200 |
| **glucose** | **mg/dL** | 96 | 0 | 400 |
| **Heart rate** | **BPM** | 4 | 0 | 200 |
| **platelets** | **10^9/L** | 96 | 0 | 800 |
| **potassium** | **mmol/L** | 96 | 2 | 7 |
| **sodium** | **mmol/L** | 96 | 105 | 200 |
| **WBC** | **10^9/L** | 96 | 0 | 40 |

S3 Table: Distribution of values: median and interquartile range of selected medical signs in different populations

| ***Dataset*** | ***AmsterdamUMC*** | | ***eICU*** | | ***MIMIC-III*** | | ***MargheritaTre*** | |
| --- | --- | --- | --- | --- | --- | --- | --- | --- |
| *(parameters)* | *median* | *IQR* | *median* | *IQR* | *median* | *IQR* | *median* | *IQR* |
| **Serum Creatinine (mg/dL)** | 1.14 | [0.81, 1.74] | 1.24 | [0.83, 2.03] | 1.30 | [0.90, 2.10] | 1.37 | [0.92, 2.15] |
| **urine_output (ml/h)** | 75.00 | [45.00, 125.00] | 57.69 | [30.00, 102.86] | 60.00 | [35.00, 120.00] | 97.08 | [69.98-125.02] |
| **HCT**  **(%)** | 30.00 | [27.00, 34.00] | 29.20 | [25.70, 33.90] | 30.10 | [27.50, 32.90] | 29.94 | [27.51, 33.3] |
| **HGB (g/dL)** | 9.99 | [9.02, 11.12] | 9.50 | [8.40, 11.10] | 10.10 | [9.20, 11.10] | 9.97 | [9.29, 11.44] |
| **Bicarbonate (mmol/L)** | 25.50 | [21.80, 29.30] | 24.00 | [21.00, 27.80] | 24.00 | [21.00, 28.00] | 25.42 | [22.08, 28.64] |
| **BUN (mg/dL)** | 33.33 | [21.29, 52.66] | 28.00 | [17.00, 46.00] | 33.00 | [20.00, 52.00] | 35.56 | [23.88, 54.68] |
| **Chloride (mmol/dL)** | 108.00 | [103.00, 113.00] | 106.00 | [101.00, 110.00] | 105.00 | [101.00, 109.00] | 105.0 | [101.5, 108.0] |
| **Glucose (mg/dL)** | 129.73 | [111.71, 154.95] | 133.00 | [108.00, 167.00] | 123.00 | [102.00, 153.00] | 139.5 | [119.83, 160.67] |
| **Heart rate (BPM)** | 88.00 | [75.00, 100.00] | 86.00 | [74.00, 100.00] | 86.00 | [75.00, 98.00] | 85.69 | [76.6, 95.38] |
| **Platelets (10^9/L)** | 195.00 | [117.00, 304.00] | 185.00 | [122.00, 260.00] | 197.00 | [116.00, 299.00] | 189.5 | [126.17, 254.0] |
| **Potassium (mmol/L)** | 4.10 | [3.80, 4.50] | 4.00 | [3.60, 4.40] | 4.10 | [3.80, 4.50] | 4.01 | [3.77, 4.3] |
| **Sodium (mmol/L)** | 142.00 | [138.00, 146.00] | 139.00 | [136.00, 143.00] | 139.00 | [136.00, 142.00] | 142.0 | [139.17, 145.48] |
| **WBC (10^9/L)** | 11.90 | [8.70, 16.40] | 10.70 | [7.61, 14.70] | 10.80 | [7.70, 14.70] | 11.74 | [9.14, 15.18] |
|  |  |  |  |  |  |  |  |  |

S4 Table: Acquisition rate: median and interquartile range distance between consecutive measurements in different populations

| ***Dataset*** | ***AmsterdamUMC*** | | ***MIMIC-III*** | | ***eICU*** | | ***MargheritaTre*** | |
| --- | --- | --- | --- | --- | --- | --- | --- | --- |
| *(parameters)* | *median(h)* | *IQR* | *median(h)* | *IQR* | *median(h)* | *IQR* | *median(h)* | *IQR* |
| **Serum Creatinine** | 19.86 | [14.78, 23.29] | 19.17 | [14.87, 22.51] | 18.54 | [15.47, 21.25] | 23.54 | [21.12, 25.02) |
| **urine_output** | 1.38 | [1.22, 1.57] | 1.62 | [1.15, 2.34] | 1.20 | [1.09, 1.39] | 1.92 | [1.01, 2.45) |
| **HCT** | 4.72 | [3.01, 8.70] | 20.38 | [15.12, 23.85] | 13.66 | [10.59, 17.07] | 22.67 | [19.86, 24.0) |
| **HGB** | 4.35 | [2.96, 8.55] | 20.37 | [14.97, 23.83] | 16.41 | [12.69, 19.72] | 22.7 | [19.79, 24.0) |
| **bicarbonate** | 3.76 | [3.02, 4.89] | 14.37 | [9.66, 19.60] | 19.11 | [15.60, 21.96] | 8.9 | [5.33, 13.01J |
| **BUN** | 24.00 | [16.87, 40.71] | 19.28 | [14.90, 22.56] | 18.65 | [15.55, 21.33] | 23.77 | [21.0, 25.07) |
| **chloride** | 3.46 | [2.68, 5.30] | 19.11 | [14.35, 22.51] | 17.88 | [14.56, 21.10] | 24.0 | [20.98, 32.0) |
| **glucose** | 3.59 | [2.82, 5.00] | 4.68 | [3.33, 8.68] | 12.91 | [9.34, 17.31] | 24.05 | [23.42, 36.0) |
| **Heart rate** | 0.98 | [0.02, 1.00] | 0.93 | [0.74, 1.10] | 0.87 | [0.78, 0.95] | 1.68 | [0.98, 2.16) |
| **platelets** | 18.61 | [13.76, 22.76] | 21.51 | [17.58, 24.19] | 19.66 | [16.61, 22.14] | 22.66 | [19.78, 24.0) |
| **potassium** | 17.45 | [11.76, 22.77] | 17.09 | [12.29, 21.18] | 11.82 | [8.94, 15.41] | 23.24 | [20.58, 24.17) |
| **sodium** | 17.38 | [11.67, 22.76] | 17.90 | [12.71, 21.93] | 16.56 | [13.51, 19.30] | 23.22 | [20.67, 24. 12) |
| **WBC** | 20.58 | [15.38, 23.71] | 21.63 | [17.90, 24.24] | 20.41 | [17.35, 22.86] | 22.66 | [19.78, 24.0) |
|  |  |  |  |  |  |  |  |  |

## Endpoint analysis

S5 Table: Persistent-AKI Staging of ICU with moderate-severe AKI

|  | AmsterdamUMC | eICU | MIMIC-III | MargheritaTre |
| --- | --- | --- | --- | --- |
| **Transient** | 60.75% | 50.10% | 62.72% | 64.26% |
| **T72** | 5.58% | 2.24% | 6.24% | 13.00% |
| **RRT24** | 2.04% | 0.60% | 1.42% | 0.29% |
| **D48** | 0.04% | 0.19% | 0.43% | 1.37% |
| **RRT<24** | 10.77% | 2.09% | 3.69% | 1.37% |
| **D<48** | 2.47% | 1.24% | 1.42% | 7.08% |
| **RRT_NM** | 2.60% | 1.40% | 1.94% | 0.00% |
| **D_NM** | 0.81% | 0.51% | 0.85% | 0.43% |
| **NM** | 8.64% | 31.80% | 9.70% | 5.63% |
| **SHORT** | 6.30% | 9.84% | 11.59% | 6.57% |
| **D<48**: Exclusion due to death before 48 hours, **RRT<24**: Exclusion due to RRT before 24 hours, **D_NM**: Death between last measurement and discharge, **RRT_NM**: RRT between last measurement and discharge, **NM**: No Measurement introducing ambiguity, **SHORT**: Aligned urine output and creatinine time series shorter than 24 hours | | | | |

S6 Table: Persistent-AKI Staging of ICU with moderate-severe AKI (post-exclusion)

|  | AmsterdamUMC | eICU | MIMIC-III | MargheritaTre |
| --- | --- | --- | --- | --- |
| **Transient** | 88.80% | 94.30% | 88.58% | 81.43% |
| **T72** | 8.15% | 4.21% | 8.82% | 16.47% |
| **RRT24** | 2.99% | 1.12% | 2.00% | 0.37% |
| **D48** | 0.06% | 0.36% | 0.60% | 1.74% |

## Population analysis

S7 Table: Main characteristics of the included population

|  | | AmsterdamUMC | | eICU | | MIMIC-III | | MargheritaTre | |
| --- | --- | --- | --- | --- | --- | --- | --- | --- | --- |
|  |  | Total | % | Total | % | Total | % | Total | % |
|  |  |  |  |  |  |  |  |  |  |
| Number of admissions |  | 1607 |  | 3562 |  | 1497 |  | 1093 |  |
| P-AKI incidence |  | 180 | 11.2% | 203 | 5.7% | 171 | 11.42% | 203 | 18.57% |
|  |  |  |  |  |  |  |  |  |  |
| Gender | F | 512 | 31.86% | 1529 | 42.93% | 658 | 43.95% | 343 |  |
|  | M | 1095 | 68.14% | 2033 | 57.07% | 839 | 56.05% | 750 | 68.6 |
|  |  |  |  |  |  |  |  |  |  |
| Age | 18-39 | 97 | 6.04% | 219 | 6.15% | 44 | 2.94% | 55 | 5.03% |
|  | 40-49 | 115 | 7.16% | 262 | 7.36% | 110 | 7.35% | 86 | 7.86% |
|  | 50-59 | 238 | 14.81% | 633 | 17.77% | 225 | 15.03% | 148 | 13.54% |
|  | 60-69 | 387 | 24.08% | 839 | 23.55% | 307 | 20.51% | 258 | 23.60% |
|  | 70-79 | 500 | 31.11% | 825 | 23.16% | 405 | 27.05% | 346 | 31.65% |
|  | 80+ | 270 | 16.80% | 782 | 21.95% | 406 | 27.12% | 200 | 18.29% |
|  |  |  |  |  |  |  |  |  |  |
| Ethnicity | Caucasian |  |  | 2844 | 79.84% | 1024 | 68.40% |  |  |
|  | African American |  |  | 398 | 11.17% | 86 | 5.74% |  |  |
|  | Hispanic |  |  | 176 | 4.94% | 31 | 2.07% |  |  |
|  | Asian |  |  | 20 | 0.56% | 21 | 1.40% |  |  |
|  | Other/Unknown |  |  | 124 | 3.48% | 335 | 22.38% |  |  |
|  |  |  |  |  |  |  |  |  |  |
| Reason for admission | Cardiovascular | 165 | 10.27% | 804 | 22.57% | 357 | 23.85% | 204 | 18.66% |
|  | Neurological | 39 | 2.43% | 174 | 4.88% | 61 | 4.07% | 205 | 18.76% |
|  | Respiratory | 194 | 12.07% | 587 | 16.48% | 126 | 8.42% | 398 | 36.41% |
|  | Sepsis | 431 | 26.82% | 705 | 19.79% | 69 | 4.61% | 128 | 11.71% |
|  | Surgery | 494 | 30.74% | 677 | 19.01% | 276 | 18.44% | 233 | 2 1.32% |
|  | Trauma | 67 | 4.17% | 251 | 7.05% | 33 | 2.20% | 208 | 19.03% |
|  | Unknown/Other | 416 | 25.89% | 827 | 23.22% | 365 | 24.38% | 0 | 0.00% |
|  |  |  |  |  |  |  |  |  |  |
| Comorbidities | Chronic Obstructive Pulmonary Disease |  |  | 309 | 8.67% | 331 | 22.11% | 106 | 9.69% |
|  | Cancer |  |  | 245 | 6.88% | 155 | 10.35% | 114 | 10.43% |
|  | Chronic kidney disease |  |  | 1196 | 33.58% | 105 | 7.01% | 129 | 11.80% |
|  | Congestive Heart failure |  |  | 403 | 11.31% | 688 | 45.96% | 111 | 10.15% |
|  | Coronary artery disease |  |  | 229 | 6.43% | 575 | 38.41% | 124 | 11.34% |
|  | Diabetes mellitus |  |  | 1012 | 28.41% | 483 | 32.26% | 602 | 55.07% |
|  | Hypertension |  |  | 573 | 16.09% | 753 | 50.30% | 253 | 23.147% |
|  |  |  |  |  |  |  |  |  |  |
|  |  |  |  |  |  |  |  |  |  |
| ICU type | Intensive Care | 416 | 25.89% | 254 | 7.13% | 378 | 25.25% | 902 | 82.53% |
|  | Medium Care | 150 | 9.33% |  |  |  |  |  |  |
|  | MC&IC | 271 | 16.86% |  |  |  |  |  |  |
|  | Coronary Care and Cardiotoracic |  |  | 270 | 7.58% | 280 | 18.70% |  |  |
|  | Cardiosurgical |  |  | 48 | 1.35% | 487 | 32.53% | 65 | 5.95% |
|  | Cardiotoracic |  |  | 102 | 2.86% |  |  |  |  |
|  | Cardiac ICU |  |  | 249 | 6.99% |  |  | 90 | 8.23% |
|  | Med-Surgical |  |  | 2150 | 60.36% |  |  |  |  |
|  | Neuro |  |  | 162 | 4.55% |  |  | 36 | 3.29% |
|  | Surgical |  |  | 327 | 9.18% | 252 | 16.83% |  |  |
|  | Trauma-Surgical |  |  |  |  | 100 | 6.68% |  |  |

S8 Table: Persistent-AKI incidence in different subpopulations

|  | | AmsterdamUMC | | eICU | | MIMIC-III | | MargheritaTre | |
| --- | --- | --- | --- | --- | --- | --- | --- | --- | --- |
|  |  | Total | Incidence | Total | Incidence | Total | Incidence | Total | Incidence |
| Number of patients P-AKI |  | 180 |  | 203 |  | 171 |  | 203 |  |
|  |  |  |  |  |  |  |  |  |  |
| Gender | F | 52 | 10.16% | 68 | 4.45% | 80 | 12.16% | 59 | 17.20% |
|  | M | 128 | 11.69% | 135 | 6.64% | 91 | 10.85% | 144 | 19.20% |
|  |  |  |  |  |  |  |  |  |  |
| Age | 18-39 | 17 | 17.53% | 13 | 5.94% | 8 | 18.18% | 7 | 12.96% |
|  | 40-49 | 12 | 10.43% | 27 | 10.31% | 23 | 20.91% | 12 | 13.95% |
|  | 50-59 | 21 | 8.82% | 50 | 7.90% | 28 | 12.44% | 25 | 16.89% |
|  | 60-69 | 53 | 13.70% | 47 | 5.60% | 31 | 10.10% | 53 | 20.54% |
|  | 70-79 | 61 | 12.20% | 41 | 4.97% | 47 | 11.60% | 77 | 22.25% |
|  | 80+ | 16 | 5.93% | 25 | 3.20% | 34 | 8.37% | 29 | 14.50% |
|  |  |  |  |  |  |  |  |  |  |
| Ethnicity | Caucasian |  |  | 135 | 4.75% | 108 | 10.55% |  |  |
|  | African American |  |  | 44 | 11.06% | 16 | 18.60% |  |  |
|  | Hispanic |  |  | 4 | 2.27% | 6 | 19.35% |  |  |
|  | Asian |  |  | 1 | 5.00% | 3 | 14.29% |  |  |
|  | Other/Unknown |  |  | 19 | 15.32% | 38 | 11.34% |  |  |
|  |  |  |  |  |  |  |  |  |  |
| Reason for admission | Cardiovascular | 18 | 10.91% | 49 | 6.09% | 38 | 10.64% | 32 | 15.69% |
|  | Neurological | 1 | 2.56% | 12 | 6.90% | 6 | 9.84% | 16 | 7.80% |
|  | Respiratory | 33 | 17.01% | 40 | 6.81% | 17 | 13.49% | 99 | 24.87% |
|  | Sepsis | 72 | 16.71% | 43 | 6.10% | 8 | 11.59% | 32 | 25.00% |
|  | Surgery | 42 | 8.50% | 22 | 3.25% | 17 | 6.16% | 39 | 16.74% |
|  | Trauma | 2 | 2.99% | 16 | 6.37% | 3 | 9.09% | 23 | 11.06% |
|  | Unknown/Other | 45 | 10.82% | 47 | 5.68% | 48 | 13.15% | 0 |  |
|  |  |  |  |  |  |  |  |  |  |
| Comorbidities | Chronic Obstructive Pulmonary Disease |  |  | 13 | 4.21% | 36 | 10.88% | 21 | 19.81% |
|  | Cancer |  |  | 17 | 6.94% | 11 | 7.10% | 19 | 16.67% |
|  | Chronic kidney disease |  |  | 105 | 8.78% | 16 | 15.24% | 39 | 30.23% |
|  | Congestive Heart failure |  |  | 33 | 8.19% | 83 | 12.06% | 19 | 17.12% |
|  | Coronary artery disease |  |  | 11 | 4.80% | 42 | 7.30% | 25 | 20.16% |
|  | Diabetes mellitus |  |  | 66 | 6.52% | 58 | 12.01% | 49 | 19.37% |
|  | Hypertension |  |  | 44 | 7.68% | 82 | 10.89% | 112 | 18.60% |
|  |  |  |  |  |  |  |  |  |  |
|  |  |  |  |  |  |  |  |  |  |
| ICU type | Intensive Care | 128 | 10.79% | 21 | 8.27% | 55 | 14.55% | 177 | 19.62% |
|  | Medium Care | 6 | 4.00% |  |  |  |  |  |  |
|  | MC&IC | 46 | 16.97% |  |  |  |  |  |  |
|  | Coronary Care and Cardiotoracic |  |  | 24 | 8.89% | 31 | 11.07% |  |  |
|  | Cardiosurgical |  |  | 4 | 8.33% | 37 | 7.60% | 5 | 7.69% |
|  | Cardiotoracic |  |  | 6 | 5.88% |  |  |  |  |
|  | Cardiac ICU |  |  | 17 | 6.83% |  |  | 19 | 21.11% |
|  | Med-Surgical |  |  | 92 | 4.28% |  |  |  |  |
|  | Neuro |  |  | 11 | 6.79% |  |  | 2 | 5.56% |
|  | Surgical |  |  | 28 | 8.56% | 34 | 13.49% |  |  |
|  | Trauma-Surgical |  |  |  |  | 14 | 14.00% |  |  |

S9 Table: Quantile distribution of length of stay in each dataset.

|  | Q=25 | Q=50 | Q=75 |
| --- | --- | --- | --- |
| AmsterdamUMC | 73.66 h | 145.08 h | 334.80 h |
| MIMIC-III | 65.60 h | 110.17 h | 223.44 h |
| eICU | 51.46 h | 80.35 h | 127.72 h |
| MargheritaTre | 92.87 h | 185.67 h | 392.65 h |

## Feature importance

S10 Table: Comparison between number of features and number of features having positive feature importance (FI).

|  | ***Clinical Parameters*** | | | | | | | | | | | | |
| --- | --- | --- | --- | --- | --- | --- | --- | --- | --- | --- | --- | --- | --- |
|  | **Serum creatinine** | **Urine output** | **HCT** | **HGB** | **bicarbonate** | **BUN** | **chloride** | **glucose** | **Heart rate** | **platelets** | **potassium** | **sodium** | **WBC** |
| ***Full*** | 38 | 50 | 38 | 38 | 38 | 38 | 38 | 38 | 50 | 38 | 38 | 38 | 38 |
| ***FI>0*** | 27 | 19 | 15 | 10 | 16 | 22 | 17 | 16 | 20 | 12 | 11 | 10 | 13 |

## Comparison with serum creatinine-based models.

S11 Table: Models’ results, max metric on test set for creatinine-based models.

|  | **Dataset** | **ICU Stays** | **Persistent AKI Incidence** | **auROC** | **auROC IQR** | **auPR** | **auPR IQR** |
| --- | --- | --- | --- | --- | --- | --- | --- |
| **Creatinine Features** | **AmsterdamUMC** | 531 | 11.11% | 89.68 | [86.69, 92.90] | 43.72 | [29.29, 53.44] |
|  | **eICU** | 3562 | 5.70% | 92.32 | [90.91, 93.84] | 35.87 | [28.98, 40.67] |
|  | **MIMIC-III** | 495 | 11.52% | 91.52 | [89.00, 94.43] | 48.98 | [34.85, 60.16] |
|  | **MargheritaTre** | 1093 | 18.58% | 84.98 | [82.68, 87.54] | 46.41 | [39.81, 52.30] |
| **Creatinine** | **AmsterdamUMC** | 531 | 11.11% | 90.62 | [87.98, 93.39] | 45.48 | [30.81, 55.59] |
|  | **eICU** | 3562 | 5.70% | 90.58 | [89.05, 92.22] | 29.39 | [24.04, 33.86] |
|  | **MIMIC-III** | 495 | 11.52% | 92.68 | [90.34, 95.41] | 52.93 | [38.42, 64.42] |
|  | **MargheritaTre** | 1093 | 18.58% | 83.04 | [80.49, 85.82] | 45.44 | [38.79, 51.89] |

S12 Table: Performance for fixed 80% sensitivity Max Metric on test sets for creatinine-based model.

|  | **Threshold** | **Dataset** | **F1** | **Sensitivity** | **Specificity** | **PPV** | **NPV** | **PPV (10% prevalence)** |
| --- | --- | --- | --- | --- | --- | --- | --- | --- |
| **Creatinine Features** | 0.31718647 | **AmsterdamUMC** | 49.26 | 84.75 | 80.08 | 34.72 | 97.68 | 32.09 |
|  |  | **eICU** | 35.29 | 91.63 | 80.20 | 21.86 | 99.37 | 33.96 |
|  |  | **MIMIC-III** | 54.78 | 75.44 | 86.99 | 43.00 | 96.45 | 39.18 |
|  |  | **MargheritaTre** | 55.27 | 78.82 | 75.28 | 42.55 | 93.97 | 26.15 |
| **Creatinine** | 0.24026208 | **AmsterdamUMC** | 50.76 | 84.75 | 81.36 | 36.23 | 97.71 | 33.56 |
|  |  | **eICU** | 31.90 | 89.66 | 77.49 | 19.40 | 99.20 | 30.67 |
|  |  | **MIMIC-III** | 56.95 | 75.44 | 88.36 | 45.74 | 96.51 | 41.86 |
|  |  | **MargheritaTre** | 51.13 | 71.92 | 75.39 | 39.66 | 92.17 | 24.51 |

## Baseline serum creatinine

S12 Table: Models’ results, max metric on test set for different bSCr definition.

|  | | **Stays** | **Incidence** | **auROC** | **auROC IQR** | **auPR** | **auPR IQR** |
| --- | --- | --- | --- | --- | --- | --- | --- |
| **AmsterdamUMC** |  | 1607 | 11.20% | 94.39 | [93.23, 95.60] | 67.76 | [61.41, 74.48] |
|  | **OPAL** | 1284 | 10.59% | 92.84 | [91.07, 95.06] | 65.24 | [57.41, 73.33] |
|  | **MDRD** | 1388 | 12.03% | 93.66 | [92.22, 95.17] | 66.59 | [59.65, 73.59] |
|  | **ICU Admission** | 1196 | 8.53% | 95.47 | [93.94, 97.14] | 69.49 | [60.65, 79.34] |
| **eICU** |  | 3562 | 5.64% | 93.71 | [92.51, 95.02] | 44.77 | [37.06, 51.18] |
|  | **OPAL** | 2378 | 6.56% | 94.17 | [92.88, 95.62] | 49.28 | [39.67, 56.46] |
|  | **MDRD** | 3092 | 7.21% | 92.97 | [91.74, 94.35] | 50.47 | [42.44, 57.25] |
|  | **ICU Admission** | 2337 | 6.29% | 95.03 | [93.91, 96.33] | 49.35 | [40.48, 56.89] |
| **MIMIC-III** |  | 1460 | 11.78% | 94.52 | [93.31, 95.81] | 68.39 | [60.69, 75.66] |
|  | **OPAL** | 1055 | 9.95% | 94.10 | [92.27, 96.27] | 61.08 | [50.29, 70.75] |
|  | **MDRD** | 1235 | 13.28% | 92.06 | [90.33, 93.95] | 62.09 | [53.52, 70.45] |
|  | **ICU Admission** | 1037 | 9.64% | 95.37 | [93.87, 96.89] | 62.30 | [50.84, 70.94] |
| **MargheritaTre** |  | 1093 | 18.57% | 85.29 | [83.17, 87.54] | 49.78 | [42.82, 56.29] |
|  | **OPAL** | 740 | 19.46% | 84.53 | [81.85, 87.46] | 50.76 | [42.78, 58.51] |
|  | **MDRD** | 953 | 23.19% | 87.13 | [84.99, 89.41] | 59.60 | [52.52, 66.31] |
|  | **ICU Admission** | 726 | 19.70% | 84.51 | [81.64, 87.70] | 51.62 | [42.69, 59.91] |

S13 Table: Performance for fixed 80% sensitivity Max Metric for different bSCr definition.

|  | **bSCr Method** | **F1** | **Sensitivity** | **Specificity** | **Precision** |
| --- | --- | --- | --- | --- | --- |
| **AmsterdamUMC** |  | 0.585009 | 0.888889 | 0.85494 | 0.435967 |
|  | **OPAL** | 0.513274 | 0.852941 | 0.825784 | 0.367089 |
|  | **MDRD** | 0.617822 | 0.834225 | 0.867322 | 0.490566 |
|  | **ICU Admission** | 0.47 | 0.921569 | 0.813528 | 0.315436 |
| **eICU** |  | 0.37816 | 0.930348 | 0.821184 | 0.23731 |
|  | **OPAL** | 0.410468 | 0.955128 | 0.810531 | 0.261404 |
|  | **MDRD** | 0.464208 | 0.877049 | 0.838271 | 0.315634 |
|  | **ICU Admission** | 0.404558 | 0.965986 | 0.811416 | 0.255856 |
| **MIMIC-III** |  | 0.597137 | 0.848837 | 0.867236 | 0.460568 |
|  | **OPAL** | 0.534819 | 0.897196 | 0.835789 | 0.380952 |
|  | **MDRD** | 0.579167 | 0.727749 | 0.859944 | 0.480969 |
|  | **ICU Admission** | 0.542857 | 0.92233 | 0.83778 | 0.384615 |
| **MargheritaTre** |  | 0.542485 | 0.73399 | 0.778652 | 0.430233 |
|  | **OPAL** | 0.547011 | 0.819444 | 0.716443 | 0.410526 |
|  | **MDRD** | 0.639132 | 0.79638 | 0.79235 | 0.533742 |
|  | **ICU Admission** | 0.547457 | 0.818182 | 0.713551 | 0.411348 |

# Algorithms

## Algorithm 1 General Feature resampling technique

| **S1 Algorithm: Feature Resamplig** | | |
| --- | --- | --- |
|  | **Input:** List of measurements of a medical sign for a patient | |
|  | **Output:** Uniform time series of the medical sign | |
|  |  | |
|  | **Step 1:** | Standardize unit of measurements as in second column of  Medical Signs analysis  S1 Table |
|  | **Step 2:** | Filter Values according to lower and upper bound as in fourth and fifth column of  Medical Signs analysis  S1 Table |
|  | **Step 3:** | Starting from ICU admission, move with time and truncate the series if two consecutive measurements have a time delta bigger than max_gap as in second column of  Medical Signs analysis  S1 Table |
|  | **Step 4:** | Repeat **Step 3** moving backward in time |
|  | **Step 5:** | Every hour on the hour is assigned with the previous measurement |

## Algorithm 2 Feature resampling technique for urine output

| **S2 Algorithm: Urine Output Resamplig** | | |
| --- | --- | --- |
|  | **Input:** List of measurements urine output volume u(t_0_), u(t_1_), u(t_2_), ... | |
|  | **Output:** Uniform time series of the urine output | |
|  |  | |
|  | **Step 1:** | urine_output(t_i_) ← u(t_i_)/( t_i_ - t_i-1_) |
|  | **Step 2:** | Apply **Step 3** and **Step 4** of S1 Algorithm 1 |
|  | **Step 3:** | Every hour on the hour is assigned with the weighted mean of the previous measurements |
